# Involvement of Toll-Like Receptors on *Helicobacter pylori*-Induced Immunity

**DOI:** 10.1371/journal.pone.0104804

**Published:** 2014-08-25

**Authors:** Romy Käbisch, Raquel Mejías-Luque, Markus Gerhard, Christian Prinz

**Affiliations:** 1 Institut für Medizinische Mikrobiologie, Immunologie und Hygiene, Technische Universität München, München, Germany; 2 Lehrstuhl für Innere Medizin 1, Universität Witten/Herdecke, Wuppertal, Germany; Charité-University Medicine Berlin, Germany

## Abstract

Dendritic cells (DCs) play a major role in the innate immune response since they recognize a broad repertoire of PAMPs mainly via Toll-like receptors (TLRs). During *Helicobacter pylori* (*H. pylori*) infection, TLRs have been shown to be important to control cytokine response particularly in murine DCs. In the present study we analyzed the effect of blocking TLRs on human DCs. Co-incubation of human DCs with *H. pylori* resulted in the release of the pro-inflammatory cytokines IL-12p70, IL-6 and IL-10. Release of IL-12p70 and IL-10 was predominantly influenced when TLR4 signaling was blocked by adding specific antibodies, suggesting a strong influence on subsequent T cell responses through TLR4 activation on DCs. Co-incubation of *H. pylori*-primed DC with allogeneic CD4^+^ T cells resulted in the production of IFN-γ and IL-17A as well as the expression of Foxp3, validating a mixed Th1/Th17 and T_reg_ response *in vitro*. Neutralization of TLR4 during *H. pylori* infection resulted in significantly decreased amounts of IL-17A and IFN-γ and reduced levels of Foxp3-expressing and IL-10-secreting T cells. Our findings suggest that DC cytokine secretion induced upon TLR4-mediated recognition of *H. pylori* influences inflammatory and regulatory T cell responses, which might facilitate the chronic bacterial persistence.

## Introduction

*Helicobacter pylori* (*H. pylori*) is a gram-negative bacillus that selectively colonizes the human gastric mucosa. Since its discovery in 1984, it has been recognized as the principal cause of peptic ulcer disease and as the main risk factor for the development of gastric cancer [1]. In order to successfully colonize the human stomach, *H. pylori* must initially overcome multiple innate and adaptive host defenses. Indeed, during acute infection, a strong gastric mucosal inflammation caused by acute neutrophilic infiltration is accompanied by *H. pylori*-specific IgM and IgA immune response as well as an infiltration of CD4^+^ and CD8^+^ to the stomach [2]. However, despite the development of an acquired immune response, *H. pylori* can persistently colonize the stomach for decades.

Moreover, in the later course of the infection increased amounts of CD4^+^/CD25^+^ regulatory T cells (T_regs_) expressing Foxp3 are observed [3]. This cell type is known to further suppress effector T cell responses, thereby enabling *H. pylori* persistence and pathology. Interestingly, the pathogenic persistence is presumably facilitated through the induction of tolerance. In this context, DCs have been show to play a crucial role. Beside their ability to enter the gastric epithelium in order to take up bacteria, DCs play an exceptional role in the regulation of T cell-mediated immune responses which are influenced by the strength of antigen presentation, intensity of costimulation [4] and by the milieu of secreted cytokines [5].

Cytokine expression in response towards pathogens is typically induced upon stimulation of specific pattern recognition receptors (PRRs). Notably, of all cells of the immune system, DCs express the broadest repertoire of PRRs, including the Nucleotide Oligomerisation Domain receptors (NLRs), the Toll-like receptors (TLRs), and several C-type lectins, such as DC-specific intercellular-adhesion-molecule-grabbing non-integrin (CD209) or Dectin-1 [6]. The best described class of PRRs is the TLR-family, consisting of ten different receptors in humans. Each of them is responsible for the detection of specific pathogen-associated molecular patterns (PAMPs), including lipopolysaccharide (LPS) (TLR4) [7], bacterial lipoproteins and lipoteichoic acids (the heterodimers TLR1/2 [8] or TLR2/6 [9]), flagellin (TLR5) [10], unmethylated CpG motifs of bacterial and viral deoxyribonucleic acid (DNA) (TLR9) [11], double-stranded ribonucleic acid (RNA) (TLR3) [12] and single-stranded viral RNA (TLR7/8) [13]. Individual TLRs trigger specific biological responses: TLR3 and TLR4 generate both, type I interferon and inflammatory cytokine responses, whereas cell surface TLR1/2, TLR2/6 and TLR5 induce mainly inflammatory cytokines [14].

An involvement of TLR signaling on the cytokine response of murine DCs has been shown already a few years ago [15]. However, there is no clear evidence how TLR activation influences intracellular DC signaling during *H. pylori* infection. In the present study we demonstrate the involvement of TLRs in inflammatory cytokine response and maturation of human DCs during *H. pylori* infection, thereby influencing adaptive immune responses.

## Methods

### Ethics Statement

DCs were generated from fresh blood samples taken from healthy volunteers for the purpose of this study as well as for serological analysis. The study was approved by the ethics committee of the Technische Universität München. All volunteers gave written informed consent.

### Bacterial strains and culture conditions

The *H. pylori* strains G27 was grown on Wilkins-Chalgren blood agar plates supplemented with 1% Dent supplement under microaerobic conditions (10% CO_2_, 5% O_2_, 85% N_2_; 37°C). For infection experiments, bacteria were harvested, resuspended in BHI medium and examined under 40x magnifications for viability.

### Leukocyte Isolation and generation of human DCs

Peripheral blood mononuclear cells (PBMCs) were isolated from heparinized venous blood from *H. pylori* negative healthy donors, after informed consent, by density gradient centrifugation with Biocoll (Biochrom, Germany). Monocytes were isolated from PBMCs by magnetic cell labeling (MACS) with the Monocyte Isolation Kit II (Miltenyi Biotec, Germany) following the manufacturer's instructions. Their purity was determined by flow cytometry staining (anti-CD14 and anti-CD45).

Immature DCs were generated by culturing monocytes in RPMI 1640 with Glutamine (Invitrogen, USA), 10% heat-inactivated FCS (Sigma, USA) and 1% Penicillin/Streptomycin, (Invitrogen, USA), 20 ng/ml human rIL-4 (Miltenyi Biotec, Germany) and 20 ng/ml human rGM-CSF (Miltenyi Biotec, Germany) for 6 days.

### Cell infection and treatments

Immature DCs were co-incubated with *H. pylori* at multiplicity of infection (MOI) 5 for 24 hours. For TLR neutralization experiments, DCs were incubated with 5 µg/ml anti-human TLR2 (Imgenex/Biomol, Germany), 5 µg/ml anti-human TLR4 (Imgenex/Biomol, Germany) or 5 µg/ml anti-human TLR5 (InvivoGen, USA) for 1 h at 37°C. Afterwards, DCs were infected with *H. pylori* G27 at MOI 5 for 24 h. Neutralization efficiency was monitored by measuring IL-6 secretion after using specific TLR-agonists. Ultrapure *E. coli* LPS (10 ng/ml) (Sigma, USA) was used as a control for TLR4 neutralization, Pam3CSK4 (0.1 µg/ml) (InvivoGen, USA) for the TLR2 blocking, and 10 ng/ml flagellin (InvivoGen, USA) as agonist for TLR5.

### Flow Cytometry

Cells were stained with Ethidium Monoazide bromide (EMA) (Anaspec, USA) for 30 min on ice for live/dead discrimination, and then resuspended in cold FACS buffer (PBS-1 % BSA). Fluorescence-labeled antibodies recognizing CD14, CD11c, HLA-DR, CD80, CD83, CD86, CD4, CD25 and Foxp3 (eBioscience, Germany) were added following manufacturer's instructions for 30 minutes at 4°C in the dark. After incubation, cells were washed and resuspended in FACS buffer before either analysis or staining of intracellular Foxp3 expression. Intracellular staining was performed by incubating the cells with Foxp3 antibody diluted in permeabilization buffer (eBioscience) according to manufacturer's protocol. After 30 minute incubation at room temperature cells were washed, resuspended in FACS buffer and analyzed. Analysis was performed with the FACS CyAn (Beckman Coulter) and the FlowJo software.

### ELISA

For determination of cytokine secretions, culture supernatants were harvested and cytokine concentrations were assessed by sandwich ELISA according to the manufacturer's instructions (eBioscience, Germany).

### CD4^+^ T cell isolation and co-cultivation with DCs

CD4^+^ T cells were separated from PBMCs by using the CD4^+^ T cell Isolation Kit II (Miltenyi Biotec, Germany). Their purity was determined by flow cytometry (anti-CD45 and anti-CD4).

Immature DCs were infected with *H pylori* at MOI 5. After 24 h, *H. pylori* was killed by supplementation of Gentamycin (0,04 mg/ml) for 2-3 h, afterwards freshly isolated CD4^+^ T cells were added to the infected DCs at 2∶1 ratio, as previously described [16]. DCs and CD4^+^ T cells were co-cultured for 3 days at 37°C in a humidified incubator containing 5% CO_2_. Foxp3 expression was analyzed by FACS and the released cytokines were measured by ELISA.

### Statistical Analysis

For statistical analysis, ANOVA test with Dunnett's correction was performed.

## Results

### *H. pylori*-mediated activation of TLR signaling influences DC maturation

The involvement of TLRs in the recognition of *H. pylori* by DCs was shown in mice [17], while little is known about their role in human cells. To investigate the effect of TLR signaling on human DC maturation upon *H. pylori* infection, we blocked the extracellular receptors TLR2, TLR4 and TLR5 with neutralizing antibodies prior *H. pylori* challenge. Maturation of DCs was characterized by analyzing the surface expression levels of the costimulatory molecule CD86 and the maturation marker CD83.

**Figure 1:**
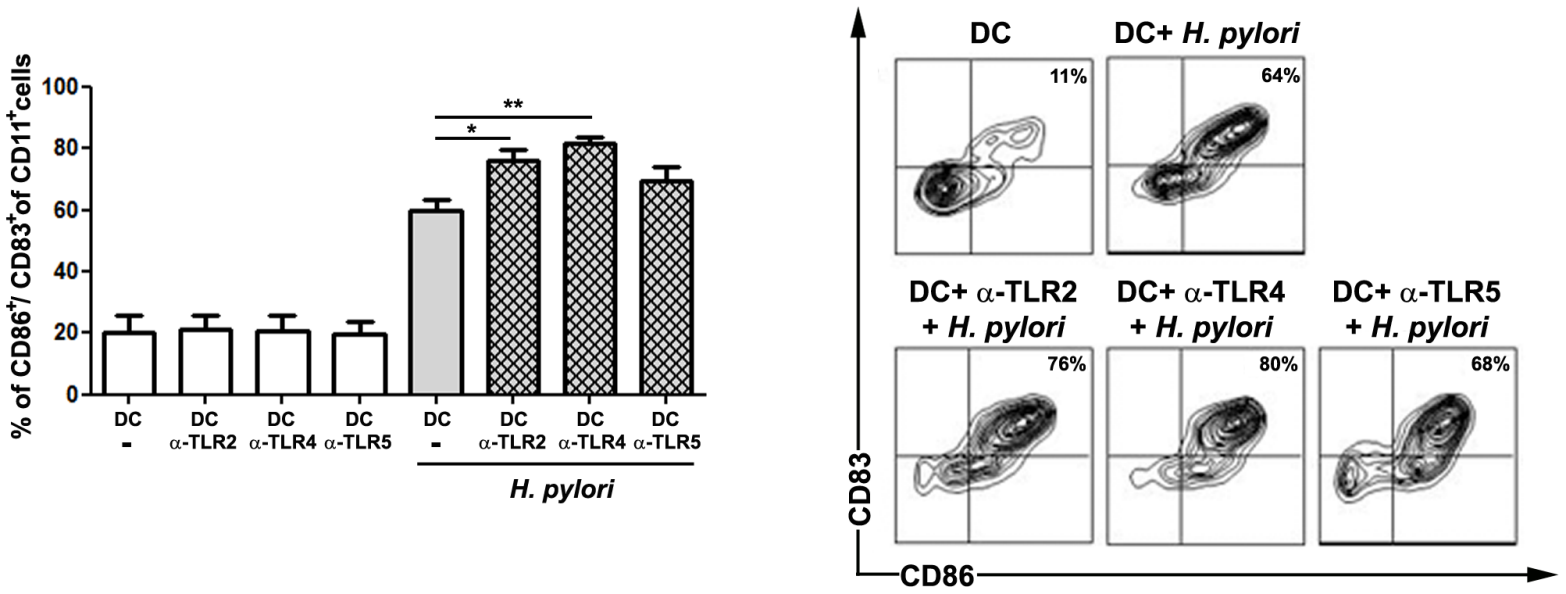
Involvement of TLRs on human DC maturation during *H. pylori* infection. Human monocyte-derived DCs were pre-incubated wit TLR-neutralizing antibodies for 1 h. Afterwards, DCs were infected with *H. pylori* G27 (MOI 5) and the levels of the costimulatory molecule CD86 and the maturation marker CD83 were analyzed by FACS on CD11c+ cells 24 h post-infection. Data are presented as mean ± SD of five independent experiments. *p≤0.05, **p≤0.005, ***p≤0.0005. Asterisks on top of bars indicate significance relative to non-neutralized, *H. pylori*-primed control cells.

*H. pylori* infection induced maturation of human DCs, leading to increased expression of CD86 and CD83 compared to uninfected control cells. Interestingly, blocking of TLR signaling during *H. pylori* infection by using neutralizing antibodies affected the maturation status of DCs: the expression of CD86 and CD83 was significantly increased when TLR2 and TLR4 were blocked, whereas no effect was detected after inhibition of TLR5 (Figure 1). These data indicate that TLR4 and to a lesser extent TLR2 signaling is involved in DC maturation during *H. pylori* infection.

### Blocking of TLR2 and TLR4 influences DC cytokine response upon *H. pylori* infection

Maturation and antigen presentation of DCs is usually accompanied by cytokine secretion that can further influence T cell polarization, thereby shaping the adaptive immune response. Therefore, we next sought to investigate the influence of TLR activation on DC cytokine secretion in response to *H. pylori* infection.

**Figure 2:**
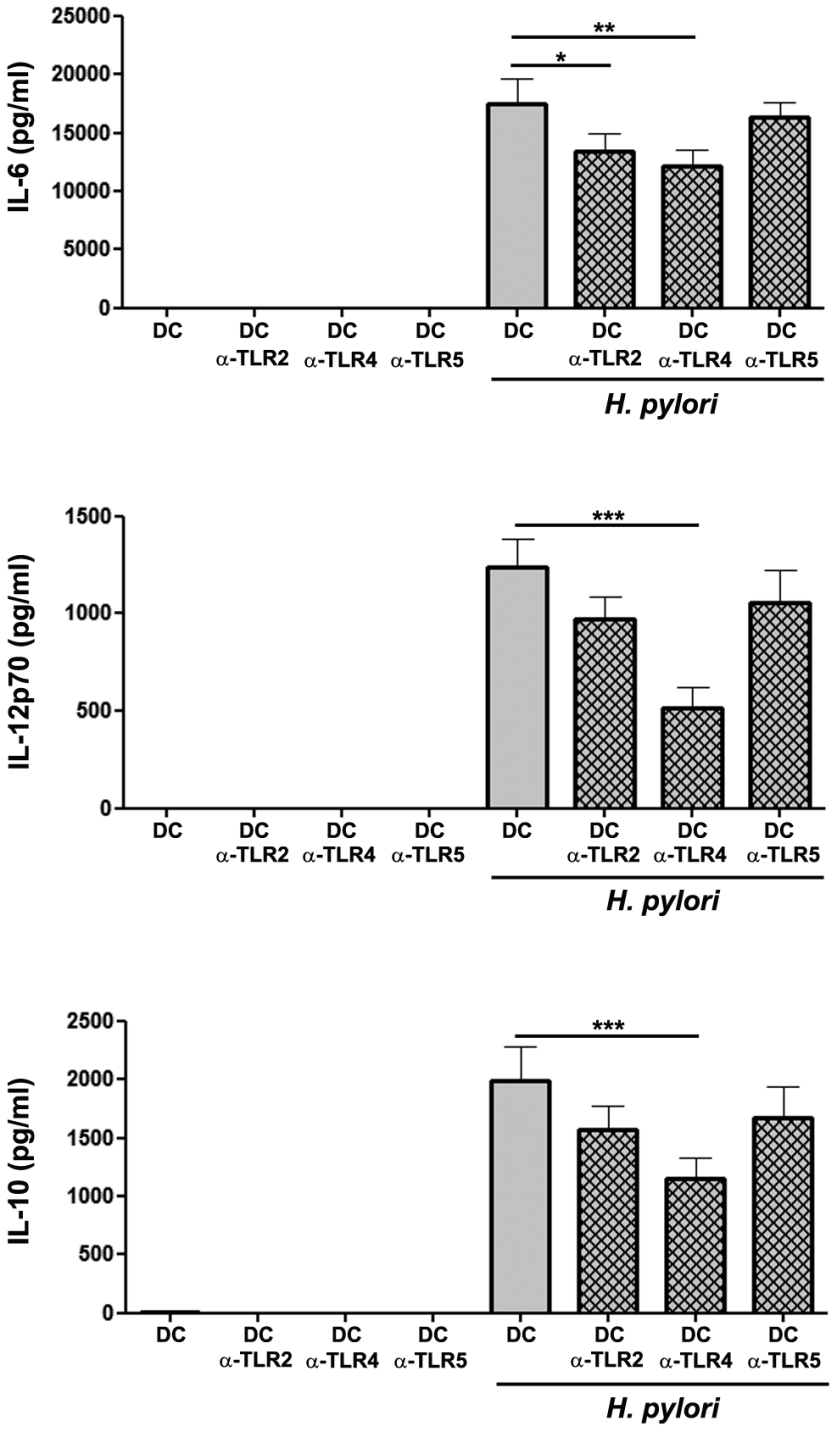
Influence of TLRs on cytokine release by human DCs in response to *H. pylori* infection. Immature human monocyte-derived DCs were pre-incubated wit TLR-neutralizing antibodies for 1 h and afterwards infected with *H. pylori* G27 (MOI 5) for 24 h. The release of IL-6, IL-12p70 and IL-10 was determined by ELISA. Data are presented as mean ± SD of six independent experiments. *p≤0.05, **p≤0.005, ***p≤0.0005. Asterisks on top of bars indicate significance relative to non-neutralized, *H. pylori*-primed control cells.

Co-incubation of human DCs with *H. pylori* resulted in the release of the pro-inflammatory cytokines IL-12p70 and IL-6 that promote T helper cell type 1 (Th1) and Th17 polarization, respectively. Similarly, IL-10, an anti-inflammatory cytokine driving T_reg_ differentiation, was also released upon *H. pylori* infection (Figure 2).

Next, *H. pylori*-mediated cytokine secretion of DCs in the presence of neutralizing antibodies was determined. Secretion of IL-6 in response to *H. pylori* was mainly blocked upon neutralization of TLR2 and TLR4 (Figure 2), but was not affected by anti-TLR5 antibodies, suggesting that TLR2 and TLR4 prime Th17 responses. The release of IL-12p70, however, was predominantly influenced when TLR4 signaling was blocked, indicating that TLR4 is the main receptor inducing Th1 responses upon *H. pylori* infection. Furthermore, the secretion of anti-inflammatory IL-10 was mainly reduced upon TLR4 neutralization, suggesting that TLR4 does not only influence inflammatory T cell responses, but also regulatory immune responses. In summary these data indicate an important role of TLR4 for the cytokine release of human DCs in response to *H. pylori* that might influence the subsequent T cell response.

### Blocking of TLRs on DCs during *H. pylori* infection influences T cell polarization

Since TLRs and specifically TLR4 deeply influence DC cytokine release, we next examined the effect of TLR inhibition on DCs on the subsequent T cell response towards *H. pylori* infection. Therefore, DCs were co-incubated with TLR-neutralizing antibodies and *H. pylori* for 24 h, afterwards CD4^+^ T cells were added and analyzed after 3 days of incubation. As a mixed Th1/Th17 response as well as a regulatory T cell response has been described in *H. pylori*-infected patients [18] [19] [20], the release of IFN-γ and IL-17A as well as the expression of Foxp3 was determined after co-culture of DCs.

**Figure 3:**
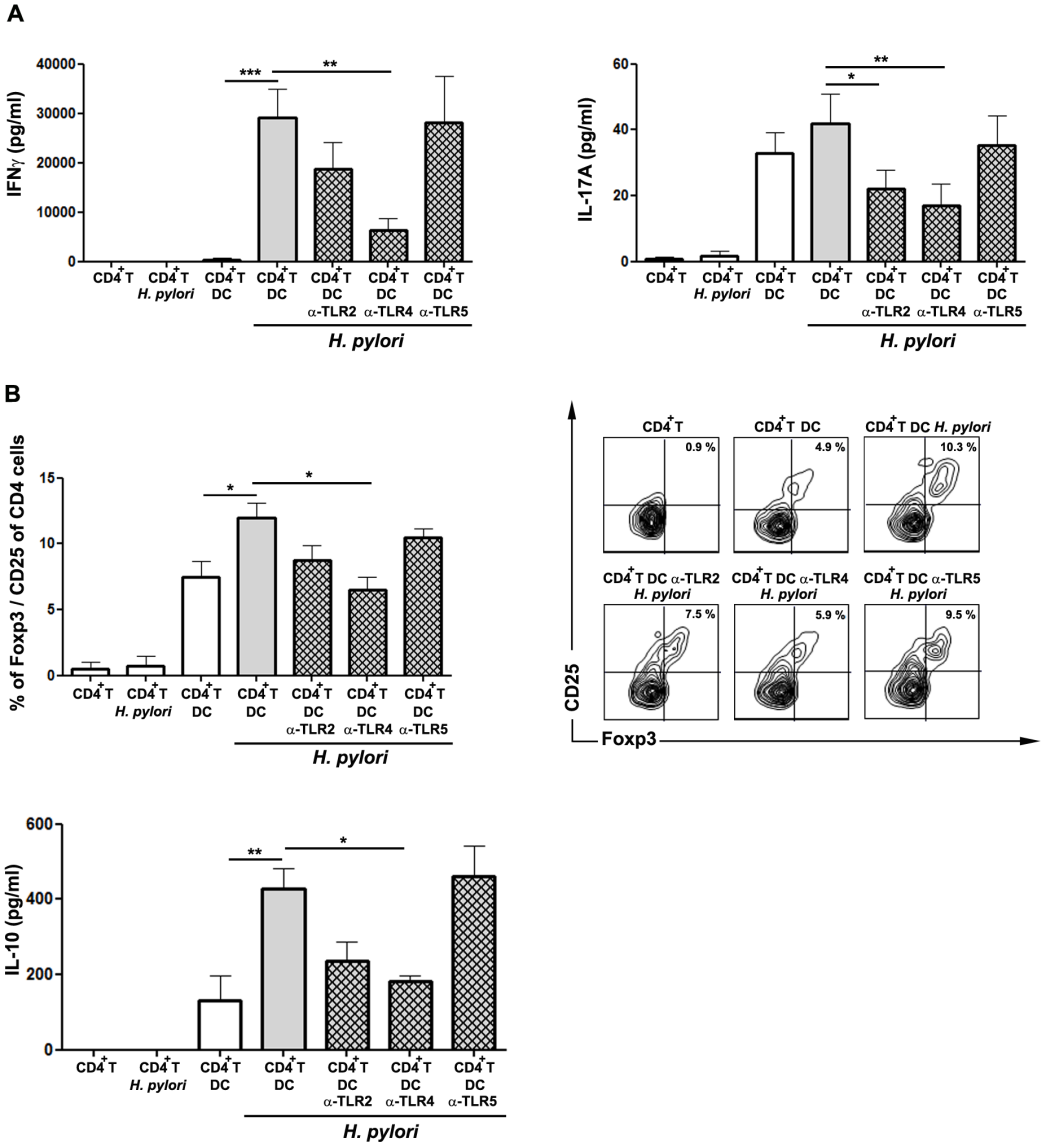
Blocking of TLRs on human DCs during *H. pylori* infection influences the adaptive T cells response. Upon neutralization of TLR signaling of human monocyte-derived DC and subsequent infection with *H. pylori* G27 (MOI 5) for 24 h, allogeneic CD4^+^ T cells were added and incubated for further 72 h. The release of (A) IFN-γ, (B) IL-17A and (C) IL-10 by co-cultured T cells was quantified by ELISA. (D) The expression of Foxp3 was determined by flow cytometry. Data are presented as mean ± S.D. of 4 independent experiments. *p≤0.05, **p≤0.005, ***p≤0.0005. Asterisks on top of bars indicate significance relative to non-neutralized, *H. pylori*-primed control cells.

Co-incubation of *H. pylori*-primed DC with allogeneic CD4^+^ T cells resulted in the production of IFN-γ and IL-17A as well as the expression of Foxp3 (Figure 3A and B), validating a mixed Th1/Th17 and T_reg_ response also *in vitro*.

Blocking of TLR2 on DCs during *H. pylori* infection induced decreased secretion of IL-17A by co-incubated CD4^+^ T cells, however the release of IFN-γ was less affected (Figure 3A). Similarly, no significant differences were observed in the expression of Foxp3 by T cells (Figure 3B). These findings confirm an involvement of *H. pylori*-mediated TLR2-activation of human DCs on subsequent Th17 activation.

In contrast, neutralization of TLR4 during *H. pylori* infection, which led to reduced secretion of IL-6, IL-12p70 as well as IL-10 by human DCs, resulted in significantly decreased amounts of IL-17A and IFN-γ (Figure 3A). Furthermore, also the levels of Foxp3-expressing and IL-10-secreting T cells were reduced (Figure 3B), validating that DC cytokine secretion induced upon TLR4-mediated recognition of *H. pylori* deeply influences inflammatory and regulatory T cell responses that might facilitate the pathogenic persistence of *H. pylori*.

## Discussion

The adaptive immune response against bacteria is activated and regulated by several innate immune cells. However, especially during *H. pylori* infection, DCs seem to play a special role in the initiation of adaptive immune responses, since increased numbers of activated DCs have been detected in the lamina propria of *H. pylori*-infected individuals [21], while in murine models DCs have been shown to induce *H. pylori*–specific adaptive immune responses [22].

Several studies have pointed out a crucial role of TLR in the context of *H. pylori* infection and gastric disease [23] [24] [25]. Moreover, we showed that TLR-mediated recognition of *H. pylori* influences murine DC activation and cytokine secretion [26], however little is known in human DCs. We analyzed the involvement of TLR signaling in human DCs upon *H. pylori* infection by blocking the receptors with neutralizing antibodies and we found that distinct TLRs induce different cytokine secretion. While the release of IL-6 by DCs upon *H. pylori* stimulation was affected by the blockage of TLR2, TLR4 and TLR5, the release of IL-12p70 was mainly influenced by TLR4 neutralization, indicating that TLR4 but no other TLRs is essential for IL-12p70 secretion upon *H. pylori* infection. The release of IL-12p70 is synergistically enhanced by the activation of MyD88 and TRIF [26]. Importantly, TLR4 is the only receptor that can activate both, leading to the generation of inflammatory cytokines and type I interferons. The latter were shown to stimulate IL-12p70 production through an autocrine-paracrine loop [27]. In contrast, TLR2 activation can only induce the expression of messenger RNA (mRNA) encoding the p40 and p19 subunits of IL-23, but not the mRNA expression of the crucial p35 subunit of IL-12 [20].

Inhibition of TLR4 signaling does not only influence IL-12p70 secretion, but also the production of IL-10. TLR neutralization on DCs showed that TLR4 and in part TLR2 induced IL-10 secretion, suggesting that their activation might be responsible for the acquisition of a tolerogenic phenotype. Indeed, neutralization of TLR2 and TLR4 led to the restoration of DC maturation after *H. pylori* infection, indicating that the secretion of IL-10 was responsible for the weak expression of costimulatory molecules and activation markers. In line with our data, *H. pylori* was shown to induce IL-10 expression via p38 MAPK and NFκB activation downstream of DC-SIGN, TLR2 and TLR4 signaling in human DCs [28]. Furthermore, it has been shown that IL-10 is involved in the down-regulation of MHC class II and CD86 expression on the surface of DCs [29]. Similarly, our study shows that inhibited TLR4 recognition of *H. pylori* in DCs leads to increased DC maturation and decreased IL-10 production.

In *H. pylori*-infected gastric lamina propria increased levels of CD4^+^ T cells have been observed [30]. Besides IL-17-producing cells [19], IFN-γ-secreting Th1 cells represent the largest group of infiltrating CD4^+^ T cells [18]. Nevertheless, this T cell response cannot successfully clear the infection. This deficiency might be explained by immune evasion mechanisms developed by *H. pylori* triggering the expansion of T_regs_. Indeed, emerging evidence suggests that *H. pylori* also induces a regulatory T cell response against T helper cell immunity, since increased expression of Foxp3 was observed in the gastric tissue of *H. pylori*-infected individuals compared to uninfected controls [20]. *H. pylori*-specific T_regs_ have been shown to suppress IFN-γ production [31] and memory T cell responses to *H. pylori* in infected individuals (3). In mice, they were capable of inhibiting Th17 differentiation [32].

We observed that co-cultivation of *H. pylori*-infected DCs with CD4^+^ T cells caused a weak secretion of IFN-γ and IL-17A, whereas increasing amounts of Foxp3-expressing T cells accompanied by enhanced IL-10 secretion were observed, confirming previous observations [33]. Blocking of TLR4 on DCs reverted the tolerogenic phenotype of the cells, suggesting that TLR4 activation on DCs might influence the subsequent T cell response during *H. pylori* infection. Indeed, decreased amounts of T_regs_ were found after co-culture experiments in the presence of TLR4-neutralizing antibodies, as measured by changes in Foxp3 expression and reduced IL-10 secretion by co-cultured CD4^+^ T cells. In addition, T cells also secreted lower levels of IFN-γ and IL-17A upon co-culture with TLR-4 neutralized *H. pylori*-infected DCs, indicating that *H. pylori*-induced DC cytokine secretion strongly influences T cell polarization during *H. pylori* infection.

Previous studies of our own group revealed that a reduction of T_regs_ by using the specific anti-CD25 antibody PC61 induced increasing levels of IFN-γ mRNA in the gastric mucosa of treated mice that was accompanied by a strong gastritis and lower bacterial colonization [34]. However, IFN-γ-deficient mice did not achieve bacterial elimination, but showed no inflammatory symptoms, suggesting an important role for IFN-γ in gastric inflammation induced by *H. pylori* [35]. In light of our results, we speculate that dysfunctional TLR4 signaling might contribute to a reduced regulatory T cell response in *H. pylori* infected individuals, while enhanced effector T cell response mediated by the activation of other TLRs as TLR2 may lead to the damage of epithelial cells and ulcerogenesis as it has been previously described [36].
